# Optimal Dose of Epidural Dexmedetomidine Added to Ropivacaine for Epidural Labor Analgesia: A Pilot Study

**DOI:** 10.1155/2017/7924148

**Published:** 2017-06-01

**Authors:** Zhang Wangping, Ren Ming

**Affiliations:** Department of Anesthesiology, Affiliated Women and Children's Hospital of Jiaxing University, Jiaxing, China

## Abstract

**Background:**

Dexmedetomidine combined with local anesthetics can decrease the concentration of epidural ropivacaine. However, the optimal dose of epidural dexmedetomidine combined with ropivacaine for labor analgesia is still uncertain. This study investigated the effect of adding different dose of epidural dexmedetomidine to ropivacaine during epidural labor analgesia.

**Methods:**

One hundred women were randomly assigned to one of the four groups (Groups A, B, C, and D received 0.25, 0.5, 0.75, and 1 *μ*g/ml of dexmedetomidine plus 0.1% ropivacaine, resp.). The onset of epidural anesthesia and stages of labor were studied, and pain was assessed using a visual analogue scale (VAS). Hemodynamic parameters and fetal heart rate were monitored. Apgar scores and umbilical artery pH were recorded. The side effects, if any, were recorded also.

**Results:**

The addition of 0.25, 0.5, and 0.75 *μ*g/ml of dexmedetomidine to 0.1% ropivacaine provided safe and effective analgesia, but 1 *μ*g/ml of dexmedetomidine resulted in increasing incidence of motor block. The hemodynamic parameters were similar between groups (*P* > 0.05). Side effects in Group D were significantly higher than those in the other three groups (*P* < 0.05).

**Conclusions:**

When dexmedetomidine is combined with 0.1% ropivacaine, the optimal concentration of dexmedetomidine is 0.5 *μ*g/ml for epidural labor analgesia (this trial is registered with ChiCTR-OPC-16008548).

## 1. Introduction

Epidural labor analgesia is the most common technique for labor pain management. Ropivacaine has been used commonly for epidural labor analgesia, because of less motor block and stable hemodynamics. Dexmedetomidine, an *α*_2_-agonist for *α*_2_-adrenergic receptors, possesses sedative and analgesic properties without respiratory depressant effect [1] and enhances their effects without increasing the incidence of side effects when added to local anesthetic agents [2–8]. At present, dexmedetomidine, although approved for intravenous use only, has been successfully used in neuraxial block and epidural block in experimental and clinical studies with less side effects [9, 10]. However, the study of dexmedetomidine is scarce in the obstetric population, and the optimal dose of epidural dexmedetomidine combined with ropivacaine for labor analgesia is still uncertain. The present study was designed to investigate the effect of adding different dose of epidural dexmedetomidine to ropivacaine during labor analgesia.

## 2. Methods

Ethical approval for this study was provided by the Ethical Committee of Jiaxing Hospital, Jiaxing, China (Chairman Professor L. Xia) on 16 July 2016. Informed consent was signed by the parturient women. One hundred parturient women (gestational weeks ≥ 37) with ASA I or II were enrolled in this prospective, single-blinded study. Exclusion criteria were as follows: ASA grades III–V, contraindication to epidural anesthesia, and patients undergoing caesarean section. One hundred parturient women were randomly assigned to one of four groups (Group A, Group B, Group C, and Group D) by using a computer-generated list (*n* = 25).

Vitals (heart rate, blood pressure, SpO_2_, and respiratory rate) were monitored immediately after entering delivery room every 5 min till the end of labor, and venous access was established. The epidural analgesia was performed at L_2-3_ interspace by an 18-gauge Tuohy needle using the method of loss of resistance to air in left lateral position. Then, an epidural catheter was inserted 3-4 cm cephalad into epidural space. Five minutes after a test dose of 5 ml of 1% lidocaine, parturient women received 8 ml of 0.25 *µ*g/ml, 0.5 *µ*g/ml, 0.75 *µ*g/ml, and 1 *µ*g/ml of dexmedetomidine, respectively, combined with 0.1% ropivacaine as loading dose, then infusing continuously this mixed solution at rate of 8 ml/h. A bolus of 8 ml (lockout time of 15 min) was administrated when visual analogue scale (VAS) scores ≥ 7.

Onset time of analgesia, blood pressure, heart rate, umbilical artery pH, fetal heart rate abnormalities, and Apgar scores were noted and analyzed. Duration of stage of labor and blood loss were also recorded. The onset of analgesia was defined as the time between the end of the epidural injection and the absence of pain at the T10 dermatome every 60 seconds by pinprick. The efficacy of the epidural analgesia was assessed at 30 min after epidural injection by VAS score (0 = no pain, 10 = worst pain). Motor block was assessed using a modified Bromage score (0 = no motor loss, 1 = inability to flex hip, 2 = inability to flex hip and knee, and 3 = inability to flex hip, knee, and ankle). The side effects including hypotension, sedation, nausea or vomiting, uroschesis, and fetal bradycardia were also studied. Respiratory depression was defined as a decrease in SpO_2_ of <94%. Fall in systolic blood pressure and heart rate by >20% from the baseline value was defined as hypotension or bradycardia, respectively.

The level of sedation was evaluated using Ramsay level of sedation scale [11] ((1) patient anxious, agitated, or restless; (2) patient cooperative, oriented, and tranquil alert; (3) patient responding to commands; (4) asleep, but with brisk response to light glabellar tap or loud auditory stimulus; (5) asleep, sluggish response to light glabellar tap or loud auditory stimulus; (6) asleep, no response). The level of sedation was evaluated every 30 min during labor using Ramsay level of sedation scale till the parturient woman was discharged from the delivery room. Excessive sedation was defined as score greater than 4.

### 2.1. Statistical Analysis

Statistical analysis was performed with SPSS 17.0 (SPSS Inc., Chicago, USA). Numerical variables were presented as mean and standard deviation (SD). Categorical data were presented as numbers. Means normally distributed were analyzed by one-way ANOVA, nonnormally distributed means were analyzed by Mann–Whitney* U* test, and categorical data were analyzed by Chi-square test. Statistical significance was defined as *P* < 0.05.

## 3. Results

**Figure 1:**
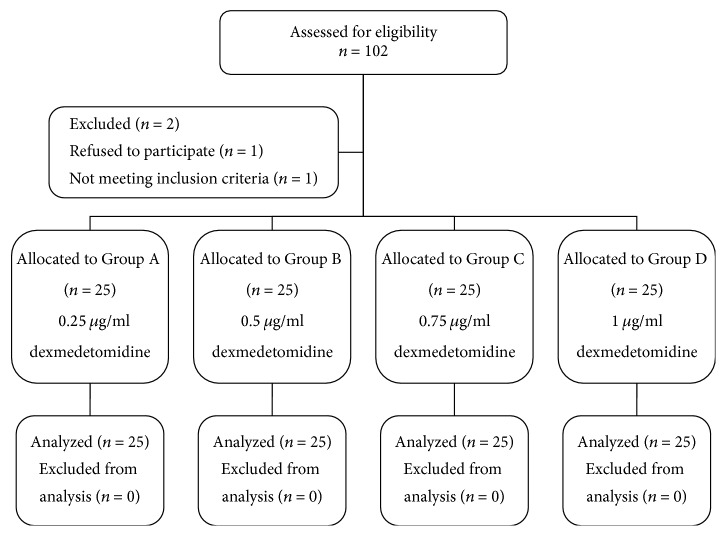
Flow diagram of study.

One hundred parturient women were enrolled in the study. No parturient woman was excluded for any reason (Figure 1). There were no differences in parturient women's demographic data including age, body weight, height, and gestation age (*P* > 0.05) (Table 1). The hemodynamic data, blood loss, mode of delivery, time of stage of labor, and onset time of analgesia were not significantly different between the four groups (*P* > 0.05) (Table 1). Neonatal Apgar score, umbilical artery pH, and umbilical artery PaO_2_ were similar in the four groups; there were no significant differences between the groups (*P* > 0.05) (Table 1).

**Figure 2:**
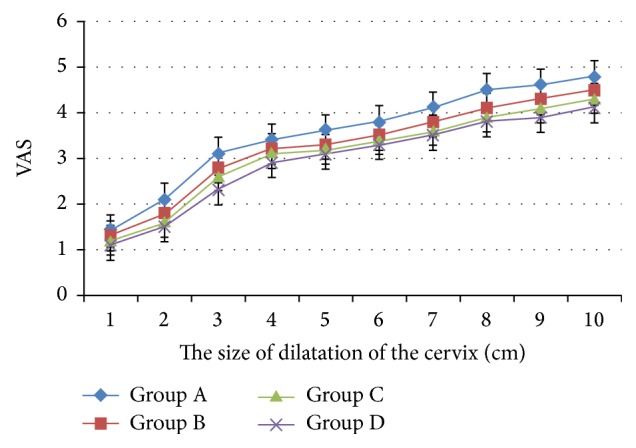
Comparison of analgesic efficacy between the groups.

The analgesic effects were enhanced with the increase of dose of dexmedetomidine in a certain range (Figure 2). There was significant difference in analgesic effects after 2 cm of cervical dilatation between Group A and Group D, but there was no significant difference in analgesic effects in Group B, Group C, and Group D. Motor block happened in Group D and Group C, but none in the other groups (Table 2). There was no significant difference in Ramsay sedation scores between the groups. Besides, there was no significant difference in the SpO_2_ and respiratory depression between the groups during labor.

Higher proportion of parturient women in four groups fall in systolic blood pressure and heart rate more than 20% of baseline value after epidural analgesia, but there were no significant differences in systolic blood pressure and heart rate between the groups. The incidence of shivering, nausea or vomiting, uroschesis, and fetal bradycardia was not significant difference between the groups.

## 4. Discussion

The ideal epidural analgesia should not only provide parturient women with satisfactory analgesia, but also reduce side effects of the mater and newborn, such as motor block, nausea and vomiting, pruritus, uroschesis, and fetal bradycardia. Dexmedetomidine has been used for enhancing the potency of epidural ropivacaine and decreasing the requirements of analgesic. In this study, we found that all of the four groups achieved good effects when four different concentrations of dexmedetomidine were added to epidural ropivacaine, and the optimal dose of epidural dexmedetomidine is 0.5 *µ*g/ml.

It is well known that fentanyl can reduce the concentration of epidural ropivacaine and decrease the requirement of ropivacaine for epidural labor analgesia. In this study, we found that the requirements of local anesthetic were reduced also when ropivacaine is combined with dexmedetomidine for epidural labor analgesia. It was according to many clinical trials in nonobstetric patients. Analgesic effects were enhanced with the increase of dose of dexmedetomidine in a certain range. Dexmedetomidine enhanced the analgesic effects without increasing the incidence of side effects when added to ropivacaine. Its mechanism of action is that it possesses selectivity, especially for an *α*_2_ receptor, which causes it to be an effective sedative and analgesic agent [12]. Compared to fentanyl, dexmedetomidine had less pruritus and less nausea and vomiting during epidural labor analgesia. It could be used safely for epidural labor analgesia. In this study, we found that analgesic efficacy in Group D was significantly better than in the other three groups, but the side effects in Group D were obviously higher than in other three groups. It was obvious that analgesic efficacy in Group A was not perfect because of lower dose of dexmedetomidine. Motor block could occur probably when 0.75 *µ*g/ml or 1 *µ*g/ml of dexmedetomidine was used for epidural labor analgesia. In this study, there was no significant difference in the SpO_2_ and umbilical artery PaO_2_ between the groups during labor. Dexmedetomidine does not cause significant respiratory depression despite providing good sedation resulting in wide safety margins [13]. In our study, Ramsay sedation scores were similar in the four groups during labor, ranging from 2 to 4, and excessive sedation score was not found. Therefore, this study could indicate that 0.5 *µ*g/ml of dexmedetomidine may be the optimal concentration for epidural labor analgesia (http://www.chictr.org.cn).

*Limitation*. In vitro study demonstrated that dexmedetomidine has the potential to enhance the frequency of uterine contractions [14]. The effect of dexmedetomidine on parturient women and fetus needs further clinical research in obstetric epidural anesthesia [15].

In summary, we could get the conclusion that 0.5 *µ*g/mL of dexmedetomidine may be the optimal concentration for parturient women in epidural labor analgesia when combined with 0.1% ropivacaine.

## Figures and Tables

**Table 1:** Data of parturient women and neonatal Apgar score.

Index	Group A	Group B	Group C	Group D	*P*
Maternal age (year)	28.4 ± 3.5	29.1 ± 4.2	27.8 ± 3.8	27.5 ± 2.9	0.521
Weight (kg)	71.3 ± 5.7	74.3 ± 6.8	69.3 ± 4.5	72.6 ± 7.1	0.258
Height (cm)	159.3 ± 3.4	161.5 ± 4.5	158.7 ± 3.6	162.2 ± 4.3	0.586
Gestational age (week)	38.7 ± 1.8	39.3 ± 2.2	38.2 ± 1.7	39.1 ± 2.1	0.321
Onset time of analgesia (min)	15.8 ± 3.6	15.4 ± 4.1	15.0 ± 3.8	14.8 ± 3.4	0.723
Time of the first stage of labor (min)	358.7 ± 86.5	372.4 ± 95.8	381.7 ± 89.4	388.2 ± 85.2	0.272
Time of the second stage of labor (min)	39.8 ± 12.6	41.2 ± 8.6	40.5 ± 9.8	42.4 ± 11.5	0.681
Blood loss (ml)	205.6 ± 19.5	198.6 ± 24.8	194.5 ± 26.7	192.2 ± 21.4	0.728
Mode of delivery (vaginal/cesarean)					
Vaginal (*n*)	22	23	24	22	0.876
Cesarean (*n*)	3	2	1	3	0.496
>20% decrease SBP (*n*)	0	0	0	0	1
>20% decrease HR (*n*)	0	0	0	0	1
Neonatal Apgar score					
At 1st min (score)	9.0 ± 0.62	8.8 ± 0.58	8.9 ± 0.56	8.7 ± 0.47	0.686
At 5th min (score)	9.7 ± 0.81	9.6 ± 0.72	9.5 ± 0.68	9.6 ± 0.75	0.564
Umbilical artery pH	7.24 ± 0.08	7.23 ± 0.06	7.21 ± 0.06	7.21 ± 0.07	0.576
Umbilical artery PaO_2_ (mmHg)	32.4 ± 6.5	31.6 ± 6.3	31.8 ± 5.9	30.2 ± 6.1	0.277

Data were presented as mean ± standard deviations or numbers. Compared between the four groups, *P* > 0.05.

**Table 2:** Side effects of anesthesia.

Index	Group A	Group B	Group C	Group D
Nausea and vomiting	0	0	0	0
Uroschesis	0	0	0	0
Hypotension	0	0	0	1
Fetal bradycardia	0	0	0	0
Respiratory depression	0	0	0	0
Bromage score (0/1/2/3)	25/0/0/0	25/0/0/0	24/1/0/0	23/2/0/0
Excessive sedation	0	0	0	0

Data were presented as numbers. Compared between the four groups, *P* > 0.05.
